# Impact of chronic ankle instability on gait loading strategy in individuals with chronic ankle instability: a comparative study

**DOI:** 10.1186/s12984-024-01478-8

**Published:** 2024-10-18

**Authors:** Omar M. Elabd, Aliaa M. Elabd, Mona S. Abd El-Azez, Mohamed M. Taha, Amira H. Mohammed

**Affiliations:** 1 Department of Orthopedics and its Surgeries, Faculty of Physical Therapy, Delta University for Science and Technology, Gamasa, Egypt; 2 Basic Science Department, Faculty of Physical Therapy, Benha University, Benha, Egypt; 3 Alumni of Faculty of Physical Therapy, Delta University for Science and Technology, Gamasa, Egypt; 4 Department of Physical Therapy for Pediatrics, Faculty of Physical Therapy, Delta University for Science and Technology, Gamasa, Egypt

**Keywords:** Chronic ankle instability, Lateral ankle sprain, Gait, Loading strategy

## Abstract

**Background:**

Lateral ankle sprains rank among the most prevalent musculoskeletal injuries, while chronic ankle instability (CAI) is its most common cascade. In addition to the conflicting results of the previous studies and their methodological flaws, the specific gait loading strategy is still not well studied.

**Purpose:**

The study aimed to investigate the fluctuations in gait loading strategy in people with chronic ankle instability compared to health control.

**Methods:**

A total of 56 male subjects participated in this study and were allocated into two groups: (A) CAI group: 28 subjects with unilateral CAI (age 24.79 ± 2.64 and BMI 26.25 ± 3.50); and (B) control group: 28 subjects without a history of ankle sprains (age 24.57 ± 1.17 and BMI 26.46 ± 2.597). Stance time, weight acceptance time, and load distribution were measured to investigate gait loading strategy.

**Results:**

The study findings revealed that the CAI group had a significant higher load over the lateral rearfoot. However, MANOVA indicates that there was no overall significant difference in gait loading strategy between the CAI and control groups. Furthermore, in terms of stance time, time of weight acceptance phase, load over medial foot, and load over lateral foot, CAI and healthy controls seemed to walk similarly.

**Conclusions:**

The findings revealed that individuals with CAI had the significant alteration in the lateral rearfoot loading, suggesting a potential compensatory mechanism to address instability during the weight acceptance phase. This could manifest a laterally deviated center of pressure and increased frontal plane inversion during the early stance phase. However, it is acknowledged that these alterations could be both the result and the origin of CAI. The study highlights the vulnerability of CAI during the early stance phase, emphasizing the need for gait reeducation as individuals return to walking as healthcare clinicians should focus on treatment modalities aimed at reducing rearfoot inversion in individuals with CAI.

## Introduction

Lateral ankle sprains rank among the most prevalent musculoskeletal injuries in both athletes and non-athletes [1, 2], while chronic ankle instability (CAI) is its common cascade, as approximately 40% of those affected go on to develop CAI [3–5]. The defining features of CAI encompass recurring ankle sprains, pain, ankle muscle weakness, limited ankle motion, and a subjective sensation of the ankle giving way, remaining for at least one year post-injury [4, 6].

Both sensorimotor and mechanical impairments could result in CAI [4, 6]. Mechanical factors involve ligamentous dysfunction due to hyperlaxity, as well as restrictions in arthrokinematics and osteokinematics [7–9]. This can also manifest even in the absence of mechanical constraints at the ankle [10]. While sensorimotor factors involve altered somatosensation, joint position sense, and reflexes, pain, ankle muscle weakness, reduced ankle range of motion (ROM), and impaired postural control [10–17].

Chronic ankle instability could result in negative health consequences such as diminished physical activity, altered movement patterns in tasks like walking, jogging, and turning, a higher risk of falls due to impaired postural control, and a higher incidence of posttraumatic ankle osteoarthritis. Also, individuals with CAI often experience functional limitations affecting daily activities, leading to poor quality of life, so CAI is a major public health issue [6, 18–27].

Gait alterations have been documented in CAI, and most of the studied parameters were spatiotemporal ones. Step length, cadence, walking speed, and single limb duration were reduced in those with CAI, while their base of support was larger. These changes in gait could be the result of patients adopting a modified gait to make up for their sensation of instability [28, 29]. Thus, these changes might have a detrimental impact on neuromuscular strategies and motor performance [30, 31].

Individuals with chronic ankle instability show altered regional activation of the peroneus longus muscle.

Researchers’ attention has been drawn to these altered neuromuscular strategies during walking, such as changes in activation patterns of ankle muscles, altered ankle kinematics, and variability in location of the center of pressure (COP), but their findings were inconsistent [3, 32–34]. In the proneus longus, tibialis anterior, and gastrocnemius, some research has found a reduction in their activity [34], while other studies have observed greater activation in the same muscles [33]. However, a recent study revealed that individuals with chronic ankle instability show altered regional activation of the peroneus longus muscle [35]. In ankle kinematics and location of COP during a stance phase, greater ankle inversion and a lateral deviation of the COP have been reported [26]. On the other hand, greater inversion was observed when running but not when walking [27]. Also, previous studies had methodological flaws due to the inclusion of subjects with bilateral CAI, ignoring that one limb influences the other and could lead to an gait alterations [36].

Besides these conflicting results and methodological flaws, the specific gait loading strategy is still not well studied. As far as we are aware, no research has thoroughly investigated how CAI affects the biomechanical aspects of gait loading strategy. In order to fill in the knowledge gap left by earlier studies and accurately specify gait loading strategy for this population, the current study used a more thorough and appropriate design that took into account the homogeneity of the sample, the elimination of bilateral CAI, and the existence of healthy controls. The study’s objectives were to investigate the changes in gait loading strategy in CAI compared to health control, hypothesizing that there was a significant difference in loading strategy between both groups. Understanding how CAI affects gait loading strategy can help design interventions to restore normal loading patterns and reduce injury risk. This knowledge can help clinicians to develop targeted rehabilitation programs, improve rehabilitation effectiveness, reduce risk of fall, and avoid complications, and improve quality of life for individuals with CAI, thereby reducing the risk of further injuries.

## Methods

### Study design

The current study, a prospective observational study, took place in compliance with the ethical principles of the 1975 Helsinki Declaration. It was held at Delta University for Science and Technology’s biomechanics lab from February 2022 to April 2023. It was registered with Clinicaltrials.gov under the identifier NCT05703828. Participants gave written agreement to a protocol approved by Ethics review Committee for Human Research at Benha University, Egypt’s Faculty of Physical Therapy (NO: PT. BU. EC. 3) before to their participation in the study.

### Participants

Participants were recruited among the students and staff of the Delta University for Science and technology, Egypt. They were referred from the outpatient physical therapy clinic and invited via advertisements on the social media. A total sample of 48 with 24 subjects in each group was calculated to detect a true and meaningful overall effect size f^2^(v) of at least 0.5, an alpha level of 0.05, and a desired power of 0.90 for 8 dependent variables using G-Power software (Version 3.1, Kiel, Germany) [37]. To ensure appropriate power due to technical issues with data recording or analysis, 56 participants were included and allocated into two groups: (A) CAI group: 28 subjects with unilateral CAI; and (B) control group: 28 subjects without a history of ankle sprains.

A minimum of one severe ankle sprain identified by an orthopedist or physiotherapist according to clinical assessment at least 1 year before the study; at least two occasions of lateral giving way; instability feeling in the ankle joint; the latest lateral ankle sprain taking place over 6 weeks before the study; the ability to support one’s entire weight on a injured limb with only minimal pain; and a score of ≤ 20 on the Arabic version of the foot function index (FFI-Arb) were requirements for inclusion in the study for participants with CAI. These criteria were established using established standards and International Ankle Consortium recommendations [7, 15, 38], with the confirmation of the validated FFI-Arb with validated specific cut-off scores at ≤ 20 [39]. Subjects in the healthy control group had no prior history of lateral ankle sprains.

Participants were excluded from the study due to any musculoskeletal or neurological conditions that could affect gait biomechanics such as major lower limb injuries, previous surgery, fractures, balance or vestibular problems, and radiated low back pain. If bilateral CAI was reported, the participant was excluded as each limb affects the other and may contribute to alteration in gait biomechanics [36].

### Assessment tools

Gait loading strategy was conducted using baropodograph system including FREEMED platform model 160 × 40 and FREESTEP software V.2.01.001 (Sensor Medica, Inc., Rome, Italy – Tel: +390774356165, Email: info@sensormedica.com) with sampling frequency of 400 Hz. This system was validated for analysis of biomechanics of gait [40, 41].

### Gait loading strategy outcomes

Stance time, weight acceptance time, and load distribution were measured to investigate loading strategy in CAI compared to healthy control group. The time when a foot touches the ground entirely or partially is known as stance time. FREESTEP software measures stance time in msec. Weight acceptance phase is the first quarter of the stance phase from the initial contact to the end of the loading response. FREESTEP software measures weight acceptance time in msec, and it’s identified when the COP moves forward away from the heel to the midfoot.

**Fig. 1:**
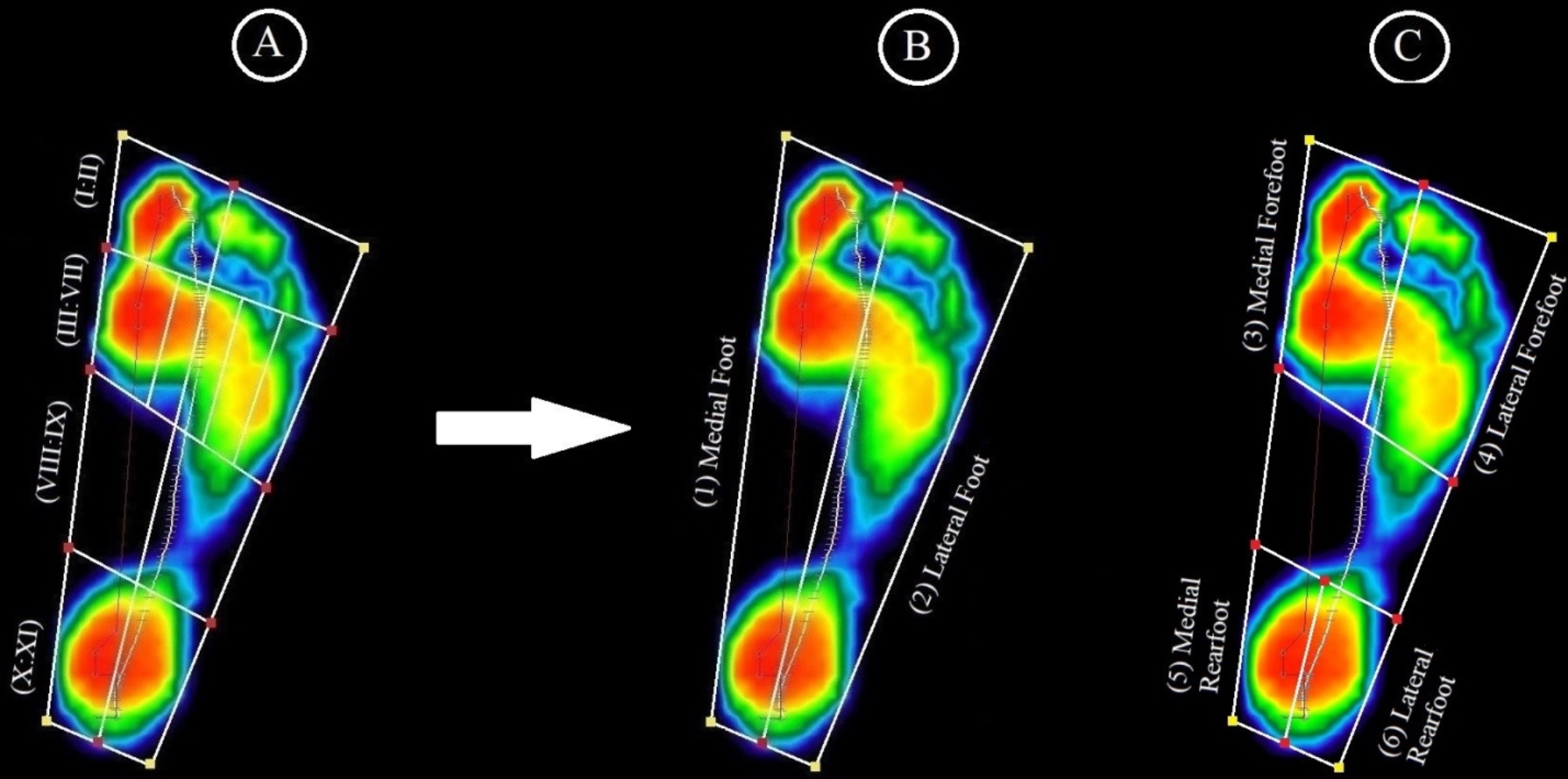
The footprint was initially split into 11 zones (**A**), however after reorganization, it was split into two zones (**B**): the medial foot (1) and the lateral foot (2) and into four zones (C): medial forefoot (3), lateral forefoot (4), medial rearfoot (5), and lateral rearfoot (6)

Regarding load distribution, the footprint is divided into 11 zones by the FREESTEP software (Fig. 1-A). To investigate loading strategy, those 11 zones were reorganized to create [1] medial foot (sum of medial zones I, III, IV, VIII, and X) [2], lateral foot (sum of lateral zones II, V-VII, IX, and XI) [3], medial forefoot (sum of zones I-III, and IV) [4], lateral forefoot (sum of zones II and V-VII) [5], medial rearfoot (zone X), and [6] lateral rearfoot (zone XI), as shown in Fig. 1-B, C [42]. There was interest in the load over these six newly constituted zones. By using the maximum load from multiple footprints that were entirely contained inside the platform’s sensing area as a reference, a percentage of the maximal load is used to represent the load over any place in the foot sole.

### Assessment procedures

The platform was set and calibrated at 10 bit auto, XY resolution at 2.5 dpi, Z resolution at 8 bit, max pressure at 150 N/cm². In order to avoid testing environment bias, participants were encouraged to get familiar with the testing atmosphere by walking barefoot over the platform for as long as necessary. Once the subject felt at ease in the laboratory setting, seven walks across the platform were captured and stored. The captured footprints were checked and verified, the successful footprints were entirely contained inside the platform’s sensing area. The unsuccessful ones that were partially inside the platform’s sensing area ones, were disabled. It’s crucial to keep in mind that walking pace directly affects the gait mechanics, meaning that modifying walking speed could change the data that was collected [43]. Therefore, walking at their self-preferred speed was adopted as the aim of the current research was to investigate naturally occurring gait loading as reflected in the biomechanical data obtained from the test process. The captured data for every participant is exported by the FREESTEP software to a PDF report. For every individual, the mean of three trials that were successful was calculated.

### Statistical analysis

By IBM SPSS Statistics (Version 26), recorded data was analyzed. The Shapiro-Wilk test was used to determine whether the recorded data along with participants’ demographic characteristics, were normal. The assumed normality of the distribution was not significantly violated. The right foot of the healthy controls was selected to be compared to the CAI foot of the CAI group. The selection was based on the non-significant differences between both sides in the control group (*p* = 0.706) which was revealed by one-way repeated multivariate analysis of variance (Paired MANOVA).

Demographic characteristics and all recorded variables of CAI and healthy control groups were calculated and presented (mean and standard deviation). MANOVA was used to compare the CAI with the control group for the demographic characteristics and the measured variables stance time, weight acceptance time, and load over (medial foot, lateral foot, medial forefoot, lateral forefoot, medial rearfoot, and lateral rearfoot). The level of significance for all tests was set at a *p*-value ≤ 0.05.

## Results

Eighty-five male participants were assessed for eligibility criteria. Fifty-six subjects met the eligibility and allocated into two groups; (A) CAI group: 28 male participants with unilateral CAI; and (B) Control group: 28 male participants without prior of ankle sprains. Subjects, and were included in the multivariate test.

Overall CAI and control groups were well-matched demographically (Wilks’ Lambda = 0.979, *F* = 0.270, and *p* = 0.896), with no significant differences in in terms of age, height, mass, and body mass index (BMI) (*p* > 0.05) (Table 1).

**Table 1:** Demographic characteristics of the subjects

Demographic	CAI *N* = 28		Control *N* = 28		*P* value
	Mean	SD	Mean	SD	
Age	24.79	2.64	24.57	1.17	0.696
Height (cm)	165.14	6.97	166.68	8.68	0.469
Weight (kg)	71.71	11.23	73.57	10.29	0.522
BMI	26.25	3.50	26.46	2.97	0.806
FFI-Arb	14.27	4.10	23.74	2.56	< 001*

The MANOVA showed there was no overall significant difference in gait loading strategy between the CAI group and healthy controls (Wilks’ Lambda = 0.847, *F* = 1.064, and *p* = 0.404). Furthermore, load over the lateral reafoot was the only variable that had a significant difference, as CAI had a higher load over the lateral reafoot in comparison to the healthy controls (*p* = 017). However, stance time, weight acceptance time, and load over medial foot, lateral foot, medial forefoot, lateral forefoot, and medial rearfoot had no significant difference between the CAI group and healthy controls (*p* > 0.05) (Table 2).

**Table 2:** Multivariate comparison for the measured variables between the CAI group and the healthy controls

Demographic	CAI *N* = 28		Control *N* = 28		*P* value
	Mean	SD	Mean	SD	
Stance time (msec)	725.11	79.63	719.32	67.59	0.771
Weight acceptance time (msec)	172.18	66.01	181.64	83.94	0.641
Load over medial foot (%)	47.31	5.08	46.24	5.17	0.441
Load over lateral foot (%)	52.58	5.23	53.70	5.07	0.423
Load over medial rearfoot (%)	16.38	2.33	16.52	3.35	0.858
Load over lateral rearfoot (%)	17.67	3.14	15.56	3.27	0.017*
Load over medial forefoot (%)	26.49	2.33	26.95	3.26	0.589
Load over lateral forefoot (%)	23.04	3.60	23.54	3.65	0.609

## Discussion

The current study aimed to investigate the impact of CAI on gait loading strategy in individuals with unilateral CAI compared to a healthy control group without prior ankle sprains. Stance time, weight acceptance time, and load over (medial foot, lateral foot, medial forefoot, lateral forefoot, medial rearfoot, and lateral rearfoot) were measured as parameters representing the gait loading strategy. The study findings provide insight into the alterations in gait loading strategy associated with CAI as the CAI group had a significant higher load over the lateral rearfoot. This suggests that CAI may change their weight distribution during early stance, specifically during the weight acceptance phase, as a possible compensatory mechanism to the instability. The well-matched demographic characteristics between the CAI and control groups enhance the internal validity of our study, ensuring that any observed differences in gait loading strategy could be attributed to the presence of CAI rather than demographic variations.

The observed increase in load over the lateral rearfoot could be explained by altered ankle mechanics, such as greater pronation-supination index, excessively lateral deviated COP, and greater frontal plane inversion from 100 msec pre-heel strike (HS) to 200 msec post-HS revealed by previous studies. Due to the changed foot position just before HS, the limb may not absorb force applied during the loading response in the best possible position. Due to the changed foot position just before HS, the limb may not absorb force applied during the loading response in the best possible position [26, 44]. These alterations could result in episodes of ankle giving way and repetitive ankle sprains, specifically if the center of mass moves outside the base of support [45]. In addition, higher inversion puts the ankle in a vulnerable position during heel strike because the early stance phase is beyond conscious control, increasing the risk of further ankle sprains and episodes of giving way [26, 44, 46]. However, it is possible that these biomechanical alterations represent the root cause of CAI rather than its effect. However, conflicting results were reported in other studies where CAI subjects were not found to have more inversion [21, 22, 27, 47] or even have greater rearfoot eversion [48].

On the other hand, MANOVA indicates that there was no overall significant difference in gait loading strategy between the CAI and control groups. Furthermore, in terms of stance time, time of weight acceptance phase, load over medial foot, and load over lateral foot, CAI and healthy controls seemed to walk with similarly. These findings indicate that most aspects of gait loading strategy are preserved in individuals with CAI. These results agree with previous studies where CAI had similar gait kinematics and kinetics with no significant differences compared to controls [21, 22, 27, 47].

This overall similarity in gait between the CAI and the healthy control group, however, is in contrast to results from other studies that reported that CAI had an increased ankle joint forefoot inversion [44, 46, 48, 49], increased lateral deviation of the COP and pronation-supination index [3, 50, 51], as well as increased vertical forces under the lateral foot [52] in comparison to the controls. The discrepancies in those results could be attributed to the data collection procedure. Current study participants were walking barefoot when data were collected. Gait mechanics differences were reported when comparing barefoot walking to shod walking [44, 45, 53–55]. This could be due to enhancing afferent feedback of proprioception and gait control in barefoot walking [21, 22].

Furthermore, this study has specific therapeutic significance as it supports earlier hypotheses that CAI may have altered proprioceptive awareness and sense of joint position [45, 56]. Medical professionals should employ a treatment approach that minimizes the possibility of experiencing recurrent ankle sprains and improve the gait mechanics by reducing the rearfoot inversion. Foot orthoses could possibly lessen these deficits [57–59].

### Limitations

This study did not look at biomechanical analyzes for lower limb’s muscle activity and joint angles for patients with CAI. Also, this study did not look at the correlation between lower limb’s muscle activity and loading strategies for patients with CAI. However, future studies on these constraints are recommended. It’s noteworthy that the gender could be a possible limitation. Therefore, the results of the current study should be cautiously applied to a female population, which may have subtle differences.

## Conclusion

The current study investigated the impact of CAI on gait loading strategy compared to healthy controls. The findings revealed that individuals with CAI had a significant alteration in the lateral rearfoot loading, suggesting a potential compensatory mechanism to address instability during the weight acceptance phase. These biomechanical alterations could be both the result and the origin of the CAI. The study highlights the vulnerability of CAI during the early stance phase, emphasizing the need for gait reeducation as individuals return to walking as healthcare clinicians should focus on treatment modalities aimed at reducing rearfoot inversion in CAI. This knowledge can help clinicians to develop targeted rehabilitation programs, improve rehabilitation effectiveness, and improve quality of life for individuals with CAI, thereby reducing the risk of further injuries. Therefore, future studies on these aspects are recommended.

## Data Availability

The corresponding author will provide the datasets used and/or analyzed for the current work upon reasonable request.

## Abbreviations

CAI: Chronic ankle instability
COP: Center of pressure
ROM: Range of motion
